# Association of Timely Outpatient Mental Health Services for Youths After Psychiatric Hospitalization With Risk of Death by Suicide

**DOI:** 10.1001/jamanetworkopen.2020.12887

**Published:** 2020-08-11

**Authors:** Cynthia A. Fontanella, Lynn A. Warner, Danielle L. Steelesmith, Guy Brock, Jeffrey A. Bridge, John V. Campo

**Affiliations:** 1 Department of Psychiatry and Behavioral Health, The Ohio State University Wexner Medical Center, Columbus; 2 University at Albany–State University of New York School of Social Welfare, Albany; 3 Department of Biomedical Informatics, The Ohio State University, Columbus; 4 Abigail Wexner Research Institute, Nationwide Children’s Hospital, Columbus, Ohio; 5 Rockefeller Neuroscience Institute, Behavioral Medicine and Psychiatry, West Virginia University, Morgantown

## Abstract

**Importance:**

Timely outpatient follow-up care after psychiatric hospitalization is an established mental health quality indicator and considered an important component of suicide prevention, yet little is known about whether follow-up care is associated with a reduced risk of suicide soon after hospital discharge.

**Objective:**

To evaluate whether receipt of outpatient care within 7 days of psychiatric hospital discharge is associated with a reduced risk of subsequent suicide among child and adolescent inpatients and examine factors associated with timely follow-up care.

**Design, Setting, and Participants:**

This population-based, retrospective, longitudinal cohort study used Medicaid data from 33 states linked with National Death Index data. The study population included all youths aged 10 to 18 years who were admitted to a psychiatric hospital from January 1, 2009, to December 31, 2013. Data analysis was completed from October 9, 2019, through May 15, 2020.

**Exposure:**

Mental health follow-up visits received within 7 days of hospital discharge.

**Main Outcomes and Measures:**

Suicides occurring in the 8 to 180 days after hospital discharge. Logistic regression modeled the association between demographic, clinical, and mental health service history factors and receipt of an outpatient visit within 7 days after discharge. Poisson regression estimated the association between suicide risk and outpatient visits within 7 days after discharge, adjusting for confounding using inverse probability of treatment weights from the logistic model.

**Results:**

Of the total 139 694 youths admitted to a psychiatric hospital, 51.9% were female, 31.1% were aged 10 to 13 years, and 68.9% were aged 14 to 18 years. A total of 56.5% of the youths received a mental health follow-up visit within 7 days of discharge, and this was associated with a significantly lower odds of suicide (adjusted relative risk, 0.44; 95% CI, 0.23-0.83; *P* = .01) during the 8 to 180 days postdischarge period. Youths with longer lengths of stay (4-5 days: adjusted odds ratio [AOR], 1.20 [95% CI, 1.17-1.24]; 6-7 days: AOR, 1.47 [95% CI, 1.43-1.52]; 8-12 days AOR, 1.75 [95% CI, 1.69-1.81]; 13-30 days: AOR, 1.71 [95% CI, 1.63-1.78]), prior outpatient mental health care (AOR, 1.58; 95% CI, 1.51-1.65), and foster care placement (AOR, 1.32; 95% CI, 1.28-1.37) were more likely to receive 7-day follow-up, whereas those who were non-Hispanic Black (AOR, 0.82; 95% CI, 0.79-0.84), were older (AOR, 0.82; 95% CI, 0.80-0.84), were medically ill (AOR, 0.77; 95% CI, 0.74-0.81), and had managed care insurance (AOR, 0.88; 95% CI, 0.87-0.91) were less likely to receive follow-up visits.

**Conclusions and Relevance:**

In this cohort study, risk of suicide during the 6 months after psychiatric hospitalization was decreased among youth who had an outpatient mental health visit within 7 days after discharge. Addressing disparities in timely continuity of care may help advance health equity agendas.

## Key Points

**Question:**

Is receipt of mental health follow-up visits within 7 days of hospital discharge associated with risk of suicide among child and adolescent inpatients, and what factors are associated with receipt of timely follow-up care?

**Findings:**

In this cohort study of 139 694 child and adolescent inpatients in the Medicaid program, mental health follow-up received within 7 days of discharge was associated with a reduced risk of suicide during the 8 to 180 days after hospital discharge. Shorter hospital stay, lack of prior mental health care, managed care, Black race, older age, and medical comorbidities were associated with delayed follow-up care.

**Meaning:**

Outpatient mental health follow-up within 7 days of psychiatric hospital discharge may be associated with a reduced risk of suicide among children and adolescents in the immediate postdischarge period.

## Teaser

This cohort study assesses whether receipt of outpatient care within 7 days of psychiatric hospital discharge is associated with a reduced risk of subsequent suicide among child and adolescent inpatients and examines factors associated with timely follow-up care.

## Introduction

A high risk of suicide and suicide attempts has been reported in the period immediately after psychiatric hospitalization.^1,2,3^ Risk of suicide attempts and death are highest immediately after psychiatric care discharge but remain elevated for months and longer. According to a recent meta-analysis,^4^ the suicide rate was 100 times the general population rate 3 months after discharge, 60 times the general population rate 3 to 12 months after discharge, and more than 30 times the general population rate for as long as 5 to 10 years thereafter. Suicide risk after psychiatric hospitalization may be increasing,^4^ suggesting that practices during and after psychiatric hospitalization may be associated with suicide risk during this period.

Timely outpatient follow-up care after psychiatric hospitalization is an established mental health quality indicator considered critical to suicide prevention.^5,6^ The National Committee on Quality Assurance and the Centers for Medicare & Medicaid Services endorse follow-up within 7 and 30 days after psychiatric hospitalization as quality indicators, but the rationale is based on clinical consensus rather than empirical evidence.^7^ Although some studies have examined the association of timely outpatient linkage with hospital readmissions,^8,9^ the association of timely outpatient care with suicide risk after inpatient psychiatric discharge has not been adequately studied.

Nationally, only approximately half of psychiatric inpatients receive outpatient mental health care in the week after discharge and approximately two-thirds receive mental health care within 1 month.^10^ Little is known about factors associated with receipt of timely postdischarge follow-up.^11^ However, given prior research on disparities in mental health utilization, demographic characteristics, such as age and race/ethnicity,^12,13^ and clinical factors^14,15^ may be associated with follow-up within 7 days after hospital discharge. This study examined receipt of outpatient care within 7 days of psychiatric inpatient discharge and the risk of suicide 180 days after discharge in a large national sample of Medicaid-insured children and adolescents and factors associated with receipt of an outpatient visit within 7 days after discharge.

## Methods

### Data Sources

This cohort study used data from the Medicaid Analytic eXtract Files (from January 1, 2009, to December 31, 2013) from 33 states obtained from the Centers for Medicare & Medicaid Services. These states were selected because of the quality and completeness of the managed care claims.^16,17^ Medicaid claims data included demographic and clinical characteristics, program eligibility, and detailed services information. To identify date and cause of death information, the Medicaid data were linked with data from the National Death Index, which provides a complete accounting of state-recorded deaths in the United States and is the most complete resource available for tracing mortality in national samples.^18^ The study was approved by The Ohio State University institutional review board with a waiver of informed consent because deidentified data were used. This study followed the Strengthening the Reporting of Observational Studies in Epidemiology (STROBE) reporting guideline.^19^

### Sample Selection

The study population included all youths (N = 142 491) aged 10 to 18 years from the 33 included states who were admitted to psychiatric hospitals between January 1, 2009, to December 31, 2013; had inpatient admissions of 1 to 30 days, were discharged home, and were continuously enrolled in Medicaid during the 180 days before the admission and the 30 days after hospital discharge. Youths who were readmitted to the hospital (n = 2797) were excluded from the analysis. There were no suicide deaths during the 7-day follow-up period. The final analytic sample included 139 694 youths.

### Measures

#### Outcome Measure

The primary outcome was suicide within 8 to 180 days of discharge. Suicide was measured by *International Statistical Classification of Diseases and Related Health Problems, Tenth Revision* codes X60 to X84, Y87.0, and *U03 (terrorism involving suicide) as the primary cause of death by the National Death Index, consistent with the US federal definition of suicide used by the Centers for Disease Control and Prevention.^20^

#### Primary Explanatory Variables

The key explanatory variable was based on Healthcare Effectiveness Data and Information Set^5^ specification for mental health follow-up in 7 days after the index psychiatric hospital discharge. An outpatient mental health visit was defined as any Medicaid-reimbursed behavioral health visit with a primary mental health diagnosis (*International Classification of Diseases, Ninth Revision, Clinical Modification* [*ICD-9-CM*] codes 290-319), including visits for psychotherapy or pharmacotherapy, partial hospitalization, rehabilitation, and other community-based services, such as case management.

#### Covariates

Demographic characteristics included age at hospital discharge (10-13 years and 14-18 years), sex (male and female), race/ethnicity (non-Hispanic White, non-Hispanic Black, Hispanic, and other as classified by Medicaid), Medicaid eligibility (poverty, disability, and foster care) and payment source (fee for service and managed care). Patients were classified into the following diagnostic groups based on primary discharge diagnoses (*ICD-9-CM* codes): depressive disorders (296.2-296.3, 298.0, 300.4, and 311), bipolar and other mood disorders (296.0, 296.4-296.8), anxiety disorders (300.0, 300.2-300.3, 293.84, 300.83, and 309.81), attention-deficit/hyperactivity disorders (314), adjustment disorders (308.3, 309.0-309.2, 309.4, and 309.9), conduct and other oppositional defiant disorders (312, 313.81), schizophrenia and other related disorders (295, 297-298), substance abuse disorders (291-292, 303-305), and other mental health disorders (290-319, not otherwise classified). Length of stay was categorized into groups based on quartiles (3, 5, and 7 days) and stays longer than 12 days. The following clinical variables were abstracted based on data from Medicaid claims from the 6 months before hospital admission: number of psychiatric comorbidities (0, 1, and ≥2), any chronic medical conditions, and deliberate self-harm (*ICD-9-CM* codes E950–E959, yes or no). The 3 following dichotomous (yes or no) variables measuring service use in the 6 months before index admission were also created: any inpatient, emergency department, or outpatient visit with a mental health diagnosis or mental health physician code.

### Statistical Analysis

Covariates including demographic and clinical characteristics and mental health service history were evaluated for association with receipt of a mental health visit within 7 days after discharge using logistic regression models. Propensity to receive a follow-up mental health visit within 7 days after discharge was calculated with a logistic regression that included all the covariates. Both unadjusted and adjusted odds ratios (AORs) were calculated along with 95% CIs and *P* values. Adjusted models included all other covariates in a multivariable model. Poisson regression accounting for the variable follow-up time for each participant modeled the association between suicide risk within 8 to 180 days of discharge and receipt of follow-up mental health visits within 7 days after discharge. Inverse probability weighting methods based on the propensity score were used to calculate adjusted relative risks (RRs), along with 95% CIs and *P* values. Inverse probability weighting diagnostic checks included assessing the balance of baseline covariates in the weighted sample using weighted standardized differences and assessing the positivity assumption.^21^ Weighted standardized differences were calculated using the packages *tableone* and *survey* in R, version 3.6.1 (R Project for Statistical Computing).^22^ Given the large sample size, we considered ORs of greater than 1.10 or less than 0.90 to be substantial from a clinical and policy perspective. Statistical significance was set at *P* = .05, and 2-sided tests were used. Data were analyzed from October 9, 2019, to May 15, 2020.

## Results

### Sample Characteristics

Of the total 139 694 youths admitted to a psychiatric hospital, 48.1% were male and 51.9% were female, 31.1% were aged 10 to 13 years, 68.9% were aged 14 to 18 years, 48.6% were non-Hispanic White, and 66.7% were enrolled in Medicaid owing to poverty. Length of stay for the initial hospitalization was most often 1 to 7 days long (77.2%). Mood disorders, such as depression (36.4%) and bipolar disorder (33.3%), were the most common primary admission diagnoses. In the 6 months before hospitalization, only 6.3% of youths had a psychiatric inpatient stay, 15.5% had a mental health emergency department visit, and 24.2% received outpatient mental health treatment. A single psychiatric diagnosis was recorded for most youths (62.5%), but 21.7% had 2 or more comorbid psychiatric conditions. Median time of Medicaid enrollment in the cohort was 173 days (interquartile range, 94-183 days; range, 16-184 days). Table 1 gives all sample characteristics.

**Table 1.** Characteristics of Youths Enrolled in Medicaid in 33 States and Discharged From a Psychiatric Hospital

Characteristic	Youths, No. (%) (N = 139 694)
Age, y	
10-13	43 499 (31.1)
14-18	96 195 (68.9)
Sex	
Female	72 537 (51.9)
Male	67 157 (48.1)
Race/ethnicity	
Non-Hispanic White	67 880 (48.6)
Non-Hispanic Black	30 489 (21.8)
Hispanic	26 023 (18.6)
Other^a^	15 302 (11.0)
Eligibility status	
Poverty	93 189 (66.7)
Disability	26 517 (19.0)
Foster care	19 988 (14.3)
Insurance type	
Fee for service	80 430 (57.6)
Managed care	59 264 (42.4)
Length of stay, d	
1-3	44 213 (31.6)
4-5	36 037 (25.8)
6-7	27 538 (19.7)
8-12	20 840 (14.9)
13-30	11 066 (7.9)
Primary discharge diagnosis	
Depression	50 794 (36.4)
Bipolar disorder or other mood disorder	46 498 (33.3)
Anxiety	4831 (3.5)
ADHD	5048 (3.6)
Substance use	5453 (3.9)
Adjustment	5553 (4.0)
Conduct or oppositional defiance	8792 (6.3)
Schizophrenia	8113 (5.8)
Other mental health disorder^b^	4612 (3.3)
Prior inpatient care	8859 (6.3)
Prior emergency department visit	21 648 (15.5)
Prior outpatient care	33 761 (24.2)
Recent self-harm	1821 (1.3)
Psychiatric comorbidities, No.	
0	87 257 (62.5)
1	22 065 (15.8)
≥2	30 372 (21.7)
Chronic medical condition	8269 (5.9)

Abbreviation: ADHD, attention-deficit/hyperactivity disorder.

^a^ Includes Native American or Alaskan, Asian, Native Hawaiian or other Pacific Islander, and more than 1 race/ethnicity.

^b^ Includes all mental health disorders coded as *International Classification of Diseases, Ninth Revision, Clinical Modification* diagnosis codes 290-319 not otherwise categorized above.

### Factors Associated With Mental Health Follow-up

A total of 56.5% of youths received a mental health visit within 7 days of inpatient discharge. Among youths receiving outpatient mental health care in the 7 days after hospitalization, most visits were for psychotherapy (29%), assessment (19.1%), case management (16.8%), and partial hospitalization (12.9%). Differences between youths who did and did not have a visit within 7 days are given in Table 2. Unweighted standardized differences corresponding to each covariate are shown in eTable 1 in the Supplement, with age, Medicaid eligibility, insurance type, length of stay, primary diagnosis, number of psychiatric comorbidities, and any prior outpatient visit having standardized differences greater than 10%. Older compared with younger youths (AOR, 0.82; 95% CI, 0.80-0.84); non-Hispanic Black and Hispanic and other racial/ethnic groups compared with non-Hispanic White (non-Hispanic Black: AOR, 0.82 [95% CI, 0.79-0.84]; other racial/ethnic groups: AOR, 0.84 [95% CI, 0.81-0.87]); and youths enrolled with managed care compared with fee for service (AOR, 0.88; 95% CI, 0.87-0.91) were less likely to receive follow-up care within 7 days of discharge. Youth enrolled in Medicaid owing to foster care compared with poverty (AOR, 1.32; 95% CI, 1.28-1.37) were more likely to receive follow-up care within 7 days of discharge. Longer lengths of stay were associated with increasing odds of receiving follow-up care within 7 days until 8 to 12 days, after which the increase in odds leveled (4-5 days: AOR, 1.20 [95% CI, 1.17-1.24]; 6-7 days: AOR, 1.47 [95% CI, 1.43-1.52]; 8-12 days: AOR, 1.75 [95% CI, 1.69-1.81]; 13-30 days: AOR, 1.71 [95% CI, 1.63-1.78]).

**Table 2.** Characteristics Associated With Follow-up Mental Health Visit Within 7 Days

Characteristic	Mental health visit within 7 d, No. (%)		OR (95% CI)	
	Yes (n = 74 632)	No (n = 65 062)	Unadjusted	Adjusted
Age, y				
10-13	25 006 (33.5)	18 493 (28.4)	1 [Reference]	1 [Reference]
14-18	49 626 (66.5)	46 569 (71.6)	0.79 (0.77-0.81)	0.82 (0.80-0.84)
Sex				
Female	39 020 (52.3)	33 517 (51.5)	1.03 (1.01-1.05)	1.05 (1.02-1.07)
Male	35 612 (47.7)	31 545 (48.5)	1 [Reference]	1 [Reference]
Race/ethnicity				
Non-Hispanic White	37 680 (50.5)	30 200 (46.4)	1 [Reference]	1 [Reference]
Non-Hispanic Black	15 547 (20.8)	14 942 (23.0)	0.83 (0.81-0.86)	0.82 (0.79-0.84)
Hispanic	13 722 (18.4)	12 301 (18.9)	0.89 (0.87-0.92)	0.91 (0.88-0.93)
Other^a^	7683 (10.3)	7619 (11.7)	0.81 (0.78-0.84)	0.84 (0.81-0.87)
Eligibility status				
Poverty	48 232 (64.6)	44 957 (69.1)	1 [Reference]	1 [Reference]
Disability	13 877 (18.6)	12 640 (19.4)	1.02 (1.00-1.05)	1.00 (0.97-1.04)
Foster care	12 523 (16.8)	7465 (11.5)	1.56 (1.52-1.61)	1.32 (1.28-1.37)
Insurance type				
Fee for service	44 950 (60.2)	35 480 (54.5)	1 [Reference]	1 [Reference]
Managed care	29 682 (39.8)	29 582 (45.5)	0.79 (0.78-0.81)	0.88 (0.87-0.91)
Length of stay, d				
1-3	19 839 (26.6)	24 374 (37.5)	1 [Reference]	1 [Reference]
4-5	19 008 (25.5)	17 029 (26.2)	1.37 (1.33-1.41)	1.20 (1.17-1.24)
6-7	16 051 (21.5)	11 487 (17.7)	1.72 (1.67-1.77)	1.47 (1.43-1.52)
8-12	13 024 (17.5)	7816 (12.0)	2.05 (1.98-2.12)	1.75 (1.69-1.81)
13-30	6710 (9.0)	4356 (6.7)	1.89 (1.81-1.97)	1.71 (1.63-1.78)
Primary discharge diagnosis				
Depression	27 742 (37.2)	23 052 (35.4)	1 [Reference]	1 [Reference]
Bipolar disorder or other mood disorder	26 955 (36.1)	19 543 (3.0)	1.15 (1.12-1.18)	1.02 (1.00-1.05)
Anxiety	2535 (3.4)	2296 (3.5)	0.92 (0.87-0.97)	0.86 (0.81-0.91)
ADHD	2539 (3.4)	2509 (3.9)	0.84 (0.79-0.89)	0.80 (0.75-0.85)
Substance use	1508 (2.0)	3945 (6.1)	0.32 (0.30-0.34)	0.35 (0.33-0.37)
Adjustment	2464 (3.3)	3089 (4.8)	0.66 (0.63-0.70)	0.75 (0.71-0.79)
Conduct or oppositional defiance	4706 (6.3)	4086 (6.3)	0.96 (0.92-1.00)	0.89 (0.85-0.93)
Schizophrenia	4430 (5.9)	3683 (5.7)	1.00 (0.95-1.05)	0.96 (0.91-1.01)
Other^b^	1753 (2.4)	2859 (4.4)	0.51 (0.48-0.54)	0.54 (0.51-0.58)
Prior inpatient care	5011 (6.7)	3848 (5.9)	1.15 (1.10-1.20)	1.19 (1.12-1.25)
Prior emergency department visit	12 381 (16.6)	9267 (14.2)	1.20 (1.16-1.23)	1.12 (1.08-1.17)
Prior outpatient care	20 922 (28.0)	12 839 (19.7)	1.58 (1.55-1.63)	1.58 (1.51-1.65)
Recent self-harm	925 (1.2)	896 (1.4)	0.90 (0.82-0.99)	0.82 (0.74-0.90)
Psychiatric comorbidities, No.				
0	44 047 (59.0)	43 210 (66.4)	1 [Reference]	1 [Reference]
1	12 109 (16.2)	9956 (15.3)	1.19 (1.16-1.23)	0.92 (0.88-0.97)
≥2	18 476 (24.8)	11 896 (18.3)	1.52 (1.48-1.57)	0.98 (0.92-1.03)
Chronic medical condition	4115 (5.5)	4154 (6.4)	0.86 (0.82-0.89)	0.77 (0.74-0.81)

Abbreviations: ADHD, attention-deficit/hyperactivity disorder; OR, odds ratio.

^a^ Includes Native American or Alaskan, Asian, Native Hawaiian or other Pacific Islander, and more than 1 race/ethnicity.

^b^ Includes all mental health disorders coded as *International Classification of Diseases, Ninth Revision, Clinical Modification* diagnosis codes 290-319 not otherwise categorized above.

Youths discharged with a primary diagnosis of depression did not have statistically significant differences from those with schizophrenia or bipolar disorder and other mood disorders in receipt of mental health visits within 7 days (schizophrenia: AOR, 0.96 [95% CI, 0.91-1.01]; bipolar and other mood disorders: AOR, 1.02 [95% CI, 1.00-1.05]). Compared with youths with a primary depression diagnosis, youths with a substance use disorder diagnosis had 65% lower odds of receiving mental health treatment within 7 days (AOR, 0.35; 95% CI, 0.33-0.37) and youths with other mental health diagnoses had a 46% lower odds (AOR, 0.54; 95% CI, 0.51-0.58). Mental health service use in the 6 months before the index hospitalization was associated with increased odds of receiving mental health services in the 7 days after discharge (inpatient: AOR, 1.19 [95% CI, 1.12-1.25]; emergency department: AOR, 1.12 [95% CI, 1.08-1.17]; outpatient: AOR, 1.58 [95% CI, 1.51-1.65]). Youths with self-harm had 18% lower odds of receiving follow-up care within 7 days (AOR, 0.82; 95% CI, 0.74-0.90), and youths with chronic medical conditions had nearly 23% lower odds (AOR, 0.77; 95% CI, 0.74-0.81).

### Association Between Follow-up and Suicide

Of the 22 youths who died by suicide within 6 months of discharge, 8 received a mental health service during the first 7 days after discharge (eTable 2 in the Supplement). Among youths who died by suicide, most were male, White, and between the ages of 14 and 18 years. Most were diagnosed with a mood disorder. Of the 22 suicides, 23% occurred from 8 to 30 days after discharge, 36% occurred from 31 to 90 days after discharge, and 41% occurred from 91 to 180 days after discharge. The unadjusted association between follow-up service and suicide was not statistically significant (RR, 0.49; 95% CI, 0.21-1.17; *P* = .11). The multivariable model shown in Table 2 was used to create propensity scores and inverse probability weights for receipt of follow-up care within 7 days. The distribution of the propensity scores is given in the eFigure in the Supplement, showing that the positivity assumption (all participants had a nonzero probability of receiving each treatment) was satisfied. The range of the propensity scores was 0.147 to 0.846, and the mean (SD) inverse probability weight from the propensity score model was 2 (0.5), with a median of 1.9 (range, 1.2-6.5). Balance diagnostics shown in eTable 1 in the Supplement indicated that all weighted standardized differences were less than 1%. The area under the receiving operating characteristic curve for the propensity score model was 0.623 (95% CI, 0.620-0.626). After the model was adjusted based on inverse weighting with propensity scores, follow-up care within 7 days was associated with a 56% lower risk of suicide (adjusted RR, 0.44; 95% CI, 0.23-0.83; *P* = .01).

## Discussion

High rates of suicide after psychiatric hospital discharge have persisted and failed to decrease for decades.^4^ In this national sample of Medicaid-covered youths admitted to psychiatric hospitals, receipt of outpatient mental health care within 7 days of discharge was associated with a significantly lower odds of suicide during the 6 months after discharge after accounting for demographic, clinical, and mental health service use characteristics. These findings support existing quality indicators and highlight the need to improve transitions from inpatient to outpatient mental health care. Despite mental health follow-up within 7 days of psychiatric discharge being a widely recognized quality expectation, nearly half of the youths in this sample failed to receive care meeting this standard. Study findings suggest a need for systematic quality improvement at the organizational level and support clinician efforts to ensure timely follow-up after psychiatric hospitalization and to educate patients and families of its importance.

Consistent with previous research conducted on samples of adults and youths,^23,24,25,26^ outpatient mental health care before inpatient hospitalization was associated with receipt of timely follow-up, underscoring the value of an established physician relationship in facilitating the transition from inpatient to ambulatory care. Conversely, patients lacking connection with mental health care before hospitalization may require more proactive clinical efforts to secure successful linkage to care and coordinate the inpatient to outpatient care transition. Assertive discharge planning, patient and family psychoeducation, and work to facilitate linkage and connectedness, such as follow-up calls, short-term case management, and bridge visits may prove to be useful for discharged patients.

Of interest, youths in this study with a history of psychiatric hospitalization or an emergency department visit before the index hospitalization were more likely to receive timely follow-up. At the individual level, experiencing recurrent hospitalizations and emergency visits may help persuade patients and families that outpatient mental health care is needed to manage symptoms, protect the patient, and prevent readmission. At the system level, prior involvement with systems of care and collaborating agencies may facilitate discharge planning and coordination of care. Involvement with an existing system of care may also be relevant to youths in foster care, who were also found in this study to be more likely to receive follow-up care in the 7 days after discharge. This finding may also reflect better monitoring and supervision by adults with behavioral health training, the involvement of case managers and the child welfare system, and better access to transportation for Medicaid-enrolled youths in foster care compared with those living in poverty.

Longer inpatients stays were also associated with a greater likelihood of outpatient mental health follow-up within 7 days of discharge, replicating prior research.^8,27,28^ In addition to allowing more time for stabilization of clinical symptoms, longer inpatient stays may offer inpatient teams more time to prepare a successful discharge plan and secure timely linkage with outpatient care. Longer lengths of hospital stay may also give outpatient facilities and professionals additional time to accommodate new or returning patients into busy and often overbooked schedules.

With regard to the association of psychiatric diagnoses with timely outpatient follow-up after psychiatric hospitalization, youths with psychiatric diagnoses other than depression, bipolar disorder, and schizophrenia were less likely to receive timely follow-up care within 7 days of hospital discharge. Of note, there was a negative association of follow-up care with the presence of a substance use disorder. Patients with substance use disorders are known to be at high risk of premature treatment termination,^29,30^ and previous research suggests that comorbid substance use disorders are associated with discontinuities of care in the transition from inpatient to ambulatory psychiatric care.^23,25^ A recent history of self-harm was also associated with a reduced likelihood of receiving timely follow-up care. This is consistent with prior studies of youths hospitalized for suicidal ideation or attempts that found about one-third failed to attend a single follow-up appointment after discharge and those who did comply typically attended only a few sessions or dropped out prematurely within 1 to 3 months of discharge.^31,32,33^ Such high rates of nonadherence with ambulatory care for suicidal youths suggest a need for focused research designed to better understand factors associated with continuity of care for this population.

Psychiatrically hospitalized youths with comorbid medical conditions were also less likely to receive timely follow-up care. These findings are consistent with prior research^34,35,36,37^ that suggests that when medical conditions co-occur, the combination is associated with elevated symptom burden, functional impairment, decreased length and quality of life, and poor adherence with treatment. Co-occurring medical conditions may be associated with increased complexities and burdens related to help seeking for affected youths and their families, suggesting that youths with comorbid psychiatric and medical conditions may benefit from more assertive discharge planning, engagement of family members, and case management services to secure outpatient follow-up visits after psychiatric discharge.

Race/ethnicity and age were associated with differences in the likelihood of receiving follow-up care. Consistent with prior research,^23,24^ older adolescents were significantly less likely than younger children to receive outpatient follow-up care meeting the 7-day quality standard. Older adolescents tend to harbor more negative attitudes about mental health treatment than younger children,^38,39^ who are less autonomous and more likely to follow parental direction. Non-Hispanic Black youths and youths in other minority groups were less likely than non-Hispanic White youths to receive follow-up within 7 days of discharge. Racial/ethnic disparities in access and quality of mental health services are well documented in the research literature,^40^ with potential factors including reduced availability of mental health specialists in minority communities, transportation, language barriers, cultural beliefs, and heightened mental health stigma among minority populations.^41^ Future quality improvement efforts may benefit by addressing such disparities, perhaps by developing and testing culturally and demographically tailored interventions to enhance continuity of care.

Similar to previous research findings that access to specialty mental health care for youths with serious emotional disturbances is more difficult to obtain under managed care,^42^ study youths enrolled in Medicaid managed care were less likely to receive timely follow-up than were youths in fee-for-service plans, even after controlling for demographic and clinical characteristics. Additional research is needed to better understand differences in access to care in association with insurance type.

Our findings provide support for the quality standard^43^ that youths discharged from inpatient psychiatric facilities should access outpatient mental health services within 7 days of discharge and suggest that adherence to this standard may be associated with reduced risk of death by suicide in the 6 months after discharge. Because 43.5% of psychiatrically hospitalized youths did not receive follow-up mental health care with 7 days of discharge, quality improvement efforts and interventions designed to improve transitions from inpatient to outpatient mental health care may be associated with a reduced short-term risk of suicide after psychiatric discharge. A number of practical interventions may improve continuity and effectiveness of care, including minimizing wait time to the first appointment, having inpatient staff clarify expectations about the role of aftercare, making appointments for patients with the aftercare agency, and reaching out techniques (eg, having the aftercare agency contact patients before the appointment and use of telephone prompts, reminder letters, and a referral coordinator).^30,44,45,46^ In addition, assertive discharge planning, shared decision-making and greater family involvement in care may also enhance service linkage after hospital discharge.

### Limitations

This study has limitations. First, our analyses focused on Medicaid-enrolled youths and may not be generalizable to privately insured or uninsured inpatients with psychiatric disorders. Nevertheless, Medicaid is the largest payer for inpatient mental health services in the US.^47^ Second, our use of claims precluded examination of other important factors that may affect receipt of follow-up care among children and adolescents, such as use of psychotropic medication, family functioning and support, caretaker perception of burden of care, and intervention strategies that hospital staff use to link patients to outpatient care. A key assumption of the propensity score model is no unmeasured confounding, and inclusion of these factors could potentially improve the discriminatory ability of the propensity score model and alter the adjusted risk estimates. We were also not able to assess the availability of services or adequacy or effectiveness of services that adolescents received. Third, limited outcome events are common problems in studies of this type, and the small number of suicide deaths may have limited statistical power. Fourth, results may not generalize to the small proportion of youths who were excluded owing to psychiatric readmission during the study period. It is possible that the subgroup of readmitted youths may represent a higher-risk group for suicide. Fifth, residual confounding may have accounted for the tendency for patients who received follow-up care to have a lower odds of suicide.

## Conclusions

In this cohort study, risk of suicide during the 6 months after psychiatric hospitalization was decreased among youth who had an outpatient mental health visit within 7 days after discharge. Addressing disparities in timely continuity of care may help advance health equity agendas.

## Notes

Supplement. **eTable 1.** Standardized Mean Differences **eTable 2.** Demographic and Clinical Characteristics of Suicide Decedents (N=22) **eFigure.** Distribution of propensity scores for receipt of a follow-up mental health (MH) visit within 7 days

## References

[1] Qin P, Nordentoft M Suicide risk in relation to psychiatric hospitalization: evidence based on longitudinal registers. Arch Gen Psychiatry. 2005;62(4):427-432. doi:10.1001/archpsyc.62.4.427 15809410

[2] Goldacre M, Seagroatt V, Hawton K Suicide after discharge from psychiatric inpatient care. Lancet. 1993;342(8866):283-286. doi:10.1016/0140-6736(93)91822-4 8101307

[3] Appleby L, Shaw J, Amos T, Suicide within 12 months of contact with mental health services: national clinical survey. BMJ. 1999;318(7193):1235-1239. doi:10.1136/bmj.318.7193.1235 10231250 PMC27859

[4] Chung DT, Ryan CJ, Hadzi-Pavlovic D, Singh SP, Stanton C, Large MM Suicide rates after discharge from psychiatric facilities: a systematic review and meta-analysis. JAMA Psychiatry. 2017;74(7):694-702. doi:10.1001/jamapsychiatry.2017.1044 28564699 PMC5710249

[5] National Committee for Quality Assurance Follow-up after hospitalization for mental illness: HEDIS measures and technical resources. Accessed January 16, 2020. https://www.ncqa.org/hedis/measures/follow-up-after-hospitalization-for-mental-illness/

[6] Luxton DD, June JD, Comtois KA Can postdischarge follow-up contacts prevent suicide and suicidal behavior? a review of the evidence. Crisis. 2013;34(1):32-41. doi:10.1027/0227-5910/a000158 22846445

[7] Hermann RC Improving Mental Healthcare: A Guide to Measurement-Based Quality Improvement. American Psychiatric Publishing; 2005.

[8] Marcus SC, Chuang C-C, Ng-Mak DS, Olfson M Outpatient follow-up care and risk of hospital readmission in schizophrenia and bipolar disorder. Psychiatr Serv. 2017;68(12):1239-1246. doi:10.1176/appi.ps.201600498 28669289

[9] Nelson EA, Maruish ME, Axler JL Effects of discharge planning and compliance with outpatient appointments on readmission rates. Psychiatr Serv. 2000;51(7):885-889. doi:10.1176/appi.ps.51.7.885 10875952

[10] National Committee for Quality Assurance Improving quality and patient experience: the state of health care quality 2013 2013. Accessed January 16, 2020. http://www.statecoverage.org/node/5079

[11] Daniel SS, Goldston DB, Harris AE, Kelley AE, Palmes GK Review of literature on aftercare services among children and adolescents. Psychiatr Serv. 2004;55(8):901-912. doi:10.1176/appi.ps.55.8.901 15292540

[12] Barksdale CL, Azur M, Leaf PJ Differences in mental health service sector utilization among African American and Caucasian youth entering systems of care programs. J Behav Health Serv Res. 2010;37(3):363-373. doi:10.1007/s11414-009-9166-2 19219552 PMC2889149

[13] Garland AF, Hough RL, Landsverk JA, Racial and ethnic variations in mental health care utilization among children in foster care. Child Serv Soc Policy Res Pract. 2000;3(3):133-146. doi:10.1207/S15326918CS0303_1

[14] Mendenhall AN Predictors of service utilization among youth diagnosed with mood disorders. J Child Fam Stud. 2012;21(4):603-611. doi:10.1007/s10826-011-9512-x

[15] Merikangas KR, He JP, Burstein M, Service utilization for lifetime mental disorders in U.S. adolescents: results of the National Comorbidity Survey-Adolescent Supplement (NCS-A). J Am Acad Child Adolesc Psychiatry. 2011;50(1):32-45. doi:10.1016/j.jaac.2010.10.006 21156268 PMC4408275

[16] Byrd V, Dodd A Assessing the usability of encounter data for enrollees in comprehensive managed care across MAX 2007-2009. Mathematica Policy Reserach; 2012 Accessed January 16, 2020. https://www.mathematica.org/our-publications-and-findings/publications/assessing-the-usability-of-encounter-data-for-enrollees-in-comprehensive-managed-care-across-max-20072009

[17] Byrd V, Dodd A Assessing the usability of encounter data for enrollees in comprehensive managed care 2010-2011. Mathematic Policy Research; 2015 Accessed January 16, 2020. https://www.mathematica.org/our-publications-and-findings/publications/assessing-the-usability-of-encounter-data-for-enrollees-in-comprehensive-managed-care-2010-2011

[18] Wojcik NC, Huebner WW, Jorgensen G Strategies for using the National Death Index and the Social Security Administration for death ascertainment in large occupational cohort mortality studies. Am J Epidemiol. 2010;172(4):469-477. doi:10.1093/aje/kwq130 20643697

[19] von Elm E, Altman DG, Egger M, Pocock SJ, Gøtzsche PC, Vandenbroucke JP; STROBE Initiative The Strengthening the Reporting of Observational Studies in Epidemiology (STROBE) statement: guidelines for reporting observational studies. Ann Intern Med. 2007;147(8):573-577. doi:10.7326/0003-4819-147-8-200710160-00010 17938396

[20] Xu J, Murphy SL, Kochanek KD, Bastian BA Deaths: final data for 2013. Natl Vital Stat Rep. 2016;64(2):1-119. 26905861

[21] Austin PC, Stuart EA Moving towards best practice when using inverse probability of treatment weighting (IPTW) using the propensity score to estimate causal treatment effects in observational studies. Stat Med. 2015;34(28):3661-3679. doi:10.1002/sim.6607 26238958 PMC4626409

[22] Lumley T Analysis of complex survey samples. J Stat Softw. 2004;9(1):1-19. doi:10.18637/jss.v009.i08

[23] Fontanella CA, Hiance-Steelesmith DL, Bridge JA, Factors associated with timely follow-up care after psychiatric hospitalization for youths with mood disorders. Psychiatr Serv. 2016;67(3):324-331. doi:10.1176/appi.ps.201500104 26620293

[24] Goldston DB, Reboussin BA, Kancler C, Rates and predictors of aftercare services among formerly hospitalized adolescents: a prospective naturalistic study. J Am Acad Child Adolesc Psychiatry. 2003;42(1):49-56. doi:10.1097/00004583-200301000-00010 12500076

[25] Olfson M, Marcus SC, Doshi JA Continuity of care after inpatient discharge of patients with schizophrenia in the Medicaid program: a retrospective longitudinal cohort analysis. J Clin Psychiatry. 2010;71(7):831-838. doi:10.4088/JCP.10m05969yel 20441730

[26] Marino L, Wissow LS, Davis M, Abrams MT, Dixon LB, Slade EP Predictors of outpatient mental health clinic follow-up after hospitalization among Medicaid-enrolled young adults. Early Interv Psychiatry. 2016;10(6):468-475. doi:10.1111/eip.12206 25639939 PMC4861685

[27] Ilgen MA, Hu KU, Moos RH, McKellar J Continuing care after inpatient psychiatric treatment for patients with psychiatric and substance use disorders. Psychiatr Serv. 2008;59(9):982-988. doi:10.1176/ps.2008.59.9.982 18757590

[28] Cuffel BJ, Held M, Goldman W Predictive models and the effectiveness of strategies for improving outpatient follow-up under managed care. Psychiatr Serv. 2002;53(11):1438-1443. doi:10.1176/appi.ps.53.11.1438 12407272

[29] Herbeck DM, Fitek DJ, Svikis DS, Montoya ID, Marcus SC, West JC Treatment compliance in patients with comorbid psychiatric and substance use disorders. Am J Addict. 2005;14(3):195-207. doi:10.1080/10550490590949488 16019970 PMC2599916

[30] Kreyenbuhl J, Nossel IR, Dixon LB Disengagement from mental health treatment among individuals with schizophrenia and strategies for facilitating connections to care: a review of the literature. Schizophr Bull. 2009;35(4):696-703. doi:10.1093/schbul/sbp046 19491314 PMC2696379

[31] Granboulan V, Roudot-Thoraval F, Lemerle S, Alvin P Predictive factors of post-discharge follow-up care among adolescent suicide attempters. Acta Psychiatr Scand. 2001;104(1):31-36. doi:10.1034/j.1600-0447.2001.00297.x 11437747

[32] Piacentini J, Rotheram-Borus MJ, Gillis JR, Demographic predictors of treatment attendance among adolescent suicide attempters. J Consult Clin Psychol. 1995;63(3):469-473. doi:10.1037/0022-006X.63.3.469 7608360

[33] Trautman PD, Stewart N, Morishima A Are adolescent suicide attempters noncompliant with outpatient care? J Am Acad Child Adolesc Psychiatry. 1993;32(1):89-94. doi:10.1097/00004583-199301000-00013 8428890

[34] Dickerson F, Brown CH, Fang L, Quality of life in individuals with serious mental illness and type 2 diabetes. Psychosomatics. 2008;49(2):109-114. doi:10.1176/appi.psy.49.2.109 18354063

[35] Egede LE Major depression in individuals with chronic medical disorders: prevalence, correlates and association with health resource utilization, lost productivity and functional disability. Gen Hosp Psychiatry. 2007;29(5):409-416. doi:10.1016/j.genhosppsych.2007.06.002 17888807

[36] Katon WJ Clinical and health services relationships between major depression, depressive symptoms, and general medical illness. Biol Psychiatry. 2003;54(3):216-226. doi:10.1016/S0006-3223(03)00273-7 12893098

[37] Stein MB, Cox BJ, Afifi TO, Belik S-L, Sareen J Does co-morbid depressive illness magnify the impact of chronic physical illness? a population-based perspective. Psychol Med. 2006;36(5):587-596. doi:10.1017/S0033291706007239 16608557

[38] Taddeo D, Egedy M, Frappier J-Y Adherence to treatment in adolescents. Paediatr Child Health. 2008;13(1):19-24. doi:10.1093/pch/13.1.19 19119348 PMC2528818

[39] Garland AF, Zigler EF Psychological correlates of help-seeking attitudes among children and adolescents. Am J Orthopsychiatry. 1994;64(4):586-593. doi:10.1037/h0079573 7847574

[40] Office of the Surgeon General (US); Center for Mental Health Services (US); National Institute of Mental Health (US) Mental health: culture, race, and ethnicity: a supplement to mental health: a report of the Surgeon General. 2001 Accessed January 16, 2020. https://www.ncbi.nlm.nih.gov/books/NBK44243/20669516

[41] Elster A, Jarosik J, VanGeest J, Fleming M Racial and ethnic disparities in health care for adolescents: a systematic review of the literature. Arch Pediatr Adolesc Med. 2003;157(9):867-874. doi:10.1001/archpedi.157.9.867 12963591

[42] Deal LW, Shiono PH, Behrman RE Children and managed health care: analysis and recommendations. Future Child. 1998;8(2):4-24. doi:10.2307/1602671 9782647

[43] Bickley H, Hunt IM, Windfuhr K, Shaw J, Appleby L, Kapur N Suicide within two weeks of discharge from psychiatric inpatient care: a case-control study. Psychiatr Serv. 2013;64(7):653-659. doi:10.1176/appi.ps.201200026 23545716

[44] Vigod SN, Kurdyak PA, Dennis C-L, Transitional interventions to reduce early psychiatric readmissions in adults: systematic review. Br J Psychiatry. 2013;202(3):187-194. doi:10.1192/bjp.bp.112.115030 23457182

[45] Klinkenberg WD, Calsyn RJ Predictors of receipt of aftercare and recidivism among persons with severe mental illness: a review. Psychiatr Serv. 1996;47(5):487-496. doi:10.1176/ps.47.5.487 8740489

[46] Boyer CA, McAlpine DD, Pottick KJ, Olfson M Identifying risk factors and key strategies in linkage to outpatient psychiatric care. Am J Psychiatry. 2000;157(10):1592-1598. doi:10.1176/appi.ajp.157.10.1592 11007712

[47] Garfield R Mental health financing in the United States: a primer. Kaiser Commission on Medicaid & the Uninsured; 2011 Accessed January 16, 2020. https://www.kff.org/medicaid/report/mental-health-financing-in-the-united-states/

