# Supplementary material for: Association of Timely Outpatient Mental Health Services for Youths After Psychiatric Hospitalization With Risk of Death by Suicide
Source: JAMA Netw Open. 2020 Aug 11;3(8):e2012887. doi: 10.1001/jamanetworkopen.2020.12887 (PMC7420244; doi:10.1001/jamanetworkopen.2020.12887)
Supplement: Supplement. — eTable 1. Standardized Mean Differences eTable 2. Demographic and Clinical Characteristics of Suicide Decedents (N=22) eFigure. Distribution of propensity scores for receipt of a follow-up mental health (MH) visit within 7 days [file jamanetwopen-3-e2012887-s001.pdf]

Supplementary Online Content

Fontanella CA, Warner LA, Steelesmith DL, Brock G, Bridge JA, Campo JV. Association of timely outpatient mental health services for youths after psychiatric hospitalization with risk of death by suicide. *JAMA Netw Open*. 2020;3(8):e2012887. doi:10.1001/jamanetworkopen.2020.12887

eTable 1. Standardized Mean Differences
eTable 2. Demographic and Clinical Characteristics of Suicide Decedents (N=22)
eFigure. Distribution of propensity scores for receipt of a follow-up mental health (MH) visit within 7 days

This supplementary material has been provided by the authors to give readers additional information about their work.

© 2020 Fontanella CA et al. *JAMA Network Open.*

eTable 1. Unweighted and Weighted Standardized Mean Differences

Variable	Levels	Unweighted Standardized Mean Difference	Weighted Standardized Mean Difference
Age Group (Years)	10-13	0.11	0.001
	14-18		
Sex	Female	0.015	0.001
	Male		
Race/Ethnicity	White	0.086	0.001
	Black		
	Hispanic		
	Other[a]		
Eligibility Status	Poverty	0.153	0.004
	Disabled		
	Foster Care		
Insurance Type	Fee for Service	0.115	0.002
	Managed Care		
Length of Stay (Days)	[0,3]	0.27	0.004
	(3,5]		
	(5,7]		
	(7,12]		
	(12,30]		
Primary Discharge Diagnosis	Depression	0.268	0.004
	Bipolar & other mood		
	Anxiety		
	ADHD		
	Substance use		
	Adjustment		
	Conduct/Oppositional Defiant		
	Schizophrenia		
	Other[b]		
Number of Psychiatric Comorbidities	0	0.17	0.005
	1		
	2+		
Chronic Medical Condition	No	0.037	0.002
	Yes		
Recent Self-Harm	No	0.012	0.001
	Yes		

© 2020 Fontanella CA et al. *JAMA Network Open.*

Variable	Levels	Unweighted Standardized Mean Difference	Weighted Standardized Mean Difference
Prior Inpatient	No	0.033	0.002
	Yes		
Prior Emergency Department	No	0.065	0.003
	Yes		
Prior Outpatient	No	0.196	0.006

Abbreviations. ADHD = Attention deficit hyperactivity disorder.

[a] Includes Native American or Alaskan, Asian, Native Hawaiian or other Pacific Islander, and more than one race. [b] Includes all mental health disorders coded as ICD-9-CM diagnosis codes 290-319 not otherwise categorized above.

© 2020 Fontanella CA et al. *JAMA Network Open.*

eTable 2. Demographic and Clinical Characteristics of Suicide Decedents (N=22)

	N	%
Age (Years)		
10-13	3	14
14-18	19	86
Sex		
Female	9	41
Male	13	59
Race/Ethnicity		
Non-Hispanic White	14	64
Other[a]	8	36
Eligibility Status		
Poverty	18	82
Other[b]	4	18
Insurance Type		
Fee for Service	11	50
Managed Care	11	50
Length of Stay (Days)		
1-8	19	86
9-30	3	14
Primary Discharge Diagnosis		
Depression	12	55
Bipolar other mood	6	27
Other[c]	4	18
Any Substance Use Disorder	3	14
Prior Mental Health Emergency Department Visit	8	36
Prior Mental Health Outpatient Visit	11	50
Number of Psychiatric Comorbidities		
0	9	41
1	4	18
2	5	23
3+	4	18
Chronic Medical Condition	1	5
Time to Suicide		
≤ 30 days	5	23
31 to ≤90 days	8	36
91+ days	9	41
Method of Suicide		
Hanging/Suffocation	15	68
Firearm	5	23
Other[d]	2	9

Note. No decedents had prior mental health inpatient visits or prior self-harm.

[a]Includes non-Hispanic black, Hispanic, Asian American, and multiple races. [b]Includes disabled and foster care. [c]Includes substance use, adjustment disorder and schizophrenia. [d]Includes jumping from high place and other/unspecified means.

© 2020 Fontanella CA et al. *JAMA Network Open.*

eFigure. Distribution of propensity scores for receipt of a follow-up mental health (MH) visit within 7 days, stratified by those who did and did not receive a follow-up visit within 7 days.

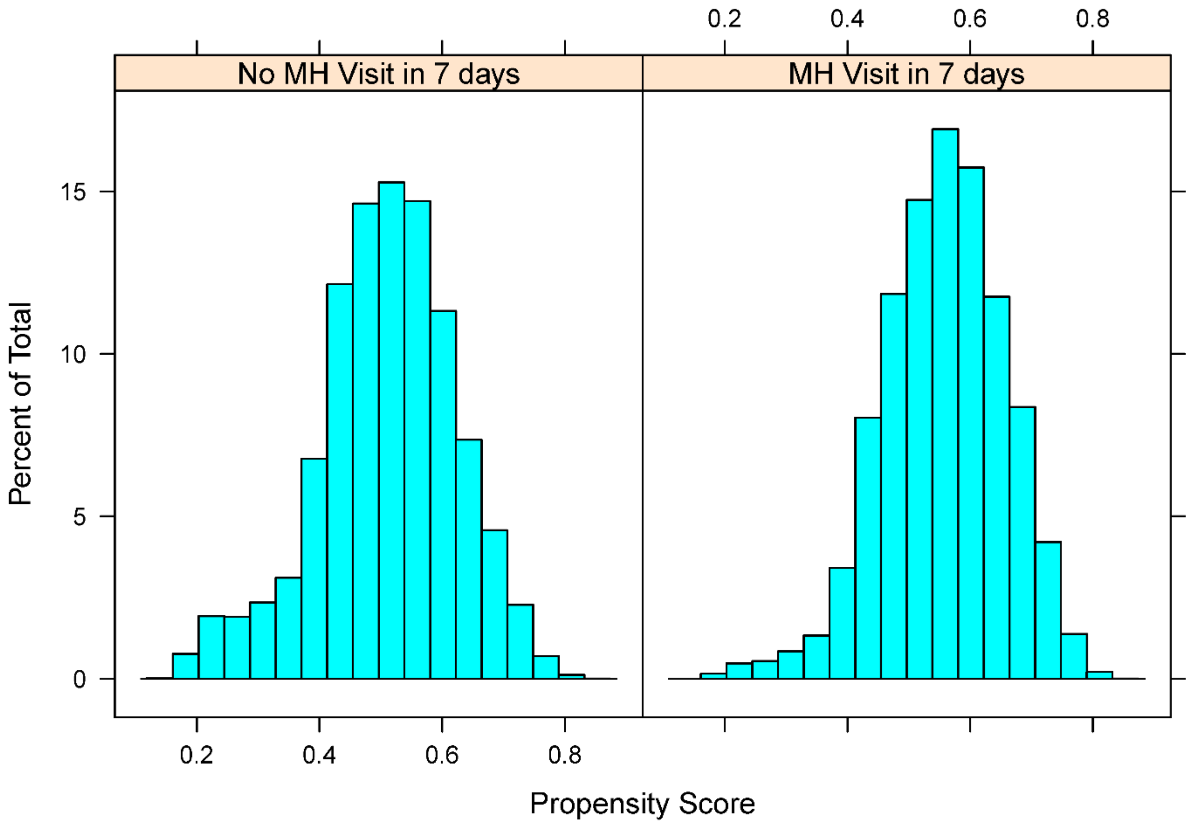

Note. The range of the propensity scores was 0.147 to 0.846.

© 2020 Fontanella CA et al. *JAMA Network Open.*
